# Early cessation of breastfeeding and the associated factors among mothers with children aged 2 to 3 years in rural Southern Ethiopia: a community-based cross-sectional study

**DOI:** 10.1186/s40795-023-00681-5

**Published:** 2023-01-31

**Authors:** Kidus Temesgen, Eshetu Andarge, Teshale Fikadu, Muluken Bekele, Yilma Chisha, Habtamu Esubalew, Temesgen Mohammed Toma

**Affiliations:** 1 School of public Health, College of Medicine and Health Science, Arba Minch University, Arba Minch, Ethiopia; 2 College of Medicine and Public health, Flinders Health and Medical Research Institute, Flinders University, Adelaide, South Australia Australia; 3 Department of nutrition and dietetics, Faculty of Public Health, Institute of Health, Jimma University, Jimma, Ethiopia; 4 Department of Public health, Arba Minch College of Health Science, Arba Minch, Ethiopia

**Keywords:** ANC, AOR, Arba Minch, Cessation of breast feeding, Children, Logistic regression, Southern Ethiopia

## Abstract

**Background:**

Breast milk is the first natural food for babies. It has nutritional, immunological, developmental, psychological, societal and environmental advantages. Failing to feed children for twenty-four months has so many negative consequences to children. Though studies have well documented the duration of breast feeding in the first six months, the proportion of women completing the recommended duration and factors associated with it has not been well investigated in rural places of Ethiopia. Therefore, this study aims to fill this gap in evidence among mothers with children aged 2 to 3 years in Arba Minch Health and Demographic Surveillance Site in 2021 E.C.

**Methods:**

A community-based cross-sectional study was conducted in all kebeles of the surveillance site by employing multi-stage sampling technique. Descriptive statistics was done to summarize findings and binary logistics regression model was used to identify factors significantly associated with early breast-feeding cessation respectively. OR with its 95% CI was obtained to quantify the degree of association between explanatory variables and early breastfeeding cessation.

**Result:**

The proportion of early cessations of breast feeding was 29.30% (25.02, 33.64%). Being from a household with no fathers ‘education or primary education [AOR=0.22; 95%CI (0.07, 0.74)] and [AOR=0.30; 95%CI (0.12, 0.76)], farmer mothers [AOR=6.40; 95%CI: (1.38, 29.74)], birth interval of less than 2 years [AOR=2.07; 95%CI: (1.03, 4.16)], and with mothers’ one or two to three antenatal care visits [AOR = 2.73; 95%CI: (1.27,5.88)] were factors significantly associated with early cessations of breast feeding.

**Conclusion and recommendation:**

The proportion of early cessations of breast feeding was high. Father’s education, being farmer, birth interval and ante natal care visit were significant factors. Health education about proper breast feeding practice and improving ante natal care attendance might improve premature cessation of breast feeding among women.

## Introduction

As a natural first food for babies, breast milk provides all the energy and nutrients that the infant needs for the first months of his life. Breast milk continues to provide up to half or more of a child’s nutritional states during the second half of the first year, and up to one-third during the second year of life [1]. Breastfeeding is the base for good nutrition, prevent, and protect children against disease. Thus, breastfeeding is the best for all children to grow and develop to their full potential [2]. Malnourishment in early years of life was mainly due to poor breast-feeding practice. During the second year of life, breast milk remains a source of vitamin A, proteins and other micronutrients that are unavailable in the usual supplementary diet, especially in low-income countries, in which breast milk can provide an average of 35 to 40% of the total energy requirements [3]. Recently, the world health organization (WHO) estimated that more than 820,000 lives of children below 5 years are lost yearly because of failing to feed children 0–23 months. Regarding its benefits in specific terms, breast feeding improves Intelligence Quotient (IQ), school attendance, child development and reduces health costs, and subsequently leads economic gains for the an individual family and a nation broadly [4].

Optimal breastfeeding of infants under two years of age greatly improves child survival in comparison to all other preventive interventions. With universal breast-feeding, 823,000 child deaths, and 20,000 maternal deaths could be averted each year, along with economic savings of US $300 billion. Likewise, breast-feeding has benefits in fewer infections, increased intelligence, probable protection against overweight and diabetes, and cancer prevention for mothers [5]. In the developing countries situations with a high burden of disease and low access to clean water and sanitation, the potential impact of optimal breastfeeding practices is especially of paramount importance [2]. Regarding intelligence, a study showed that compared to non-breastfed child, a child who has been breastfed for two years will likely to have a higher Intelligence Quotient (IQ) by 8 points [6].

Rates of breastfeeding vary widely across the world; most infants are still breast fed at 1 year in low –income countries compared with lower than 20% in many high-income countries and less than 1% in UK indicating that it is one of the few health-positive behaviors more common in poor countries than rich ones [5]. The breastfeeding cessation (before two years) varies from country to country. Pocket studies from different countries showed a varied rate of breast-feeding with a relatively high adherence in developing countries [7–12]. Breast feeding practice is more or less universal in Africa, however, there is a gap in adherence to the recommendations to the WHO; where some mothers do not initiate it timely; others do not feed it exclusively for six months, and cease feeding before two years [13].

The reasons why women avoid or stop breastfeeding range from the medical, cultural, and psychological, to physical discomfort and inconvenience [5]. Various factors affecting the early cessation of breast-feeding was documented so far from studies across the world. The factors range from the individual mother’s socio-demographic characteristics like age, educational status to different socio-cultural, breast feeding habits, knowledge, attitude and maternal and child illnesses, and obstetric factors [14–23].

Despite presence of guidelines on infant and young child feeding practices in Ethiopia [24], the duration of breastfeeding has decreased from time to time [25]. Even though few studies addressed the factors that affect duration of breast-feeding in Ethiopia [26, 27], the focus was on duration of exclusive breast-feeding than those who complete the recommended duration of breast-feeding. To the best of our search, one study conducted in Northern Ethiopia tried to assess the factors affecting cessation of breast-feeding in an urban setting at Debre Markos town [28]. Since socio-cultural variations are there in an urban and rural area and among regions, there is a need for a study in a rural area of the country.

Additionally, this study focuses on rural areas because it is usual observation to have a pregnancy shortly after childbirth, which could be as a result of a short inter-birth interval associated with the traditional belief in the society that having a child is considered as having a wealth and the low awareness, either. Thus, this might make women fall short of achieving the WHO recommendation of feeding children to 24 completed months of age. Therefore, the present study tried to fill this gap in evidence by employing a community based cross-sectional study among rural women living in Arba Minch HDSS site.

## Methods

### Study area, and period

This study was conducted in Arba Minch Health and Demographic Surveillance Site (HDSS), which is located 505 kms far to the South of Addis Ababa, capital city of Ethiopia and about 275 kms from the regional city, Hawassa. The HDSS is a longitudinal surveillance center based at Arba Minch University and collects data on maternal health, nutrition and other attributes of health from two districts nearby Arba Minch town (Arba Minch Zuria Woreda and Gacho Baba Woreda) Farming is the predominant occupation of residents in the districts [29]. According to the health office reports of the districts, the total population projected for the year 2013 E.C (2020/2021 G.C) was 207,368. The district have a total of 7 public health centers and 37 health posts. The surveillance site consists of 08 rural and one semi-urban kebeles (the smallest administrative unit in the existing Ethiopian government structure) which were selected based on generalizability to all kebeles in the woredas. In Arba Minch HDSS, there is a continuous registration and recording of maternal and newborn health services.

### Populations

Community based cross-sectional study was conducted. All mothers with children aged 2 to 3 years at Arba Minch HDSS sites, Gamo Zone, Southern Ethiopia, were source population of the current study and randomly selected mothers with children aged 2 to 3 years. Inclusion criteria of the present study was all randomly selected mothers with children aged 2 to 3 years at Arba Minch HDSS sites, Gamo Zone, Southern Ethiopia was included in the study.

### Sample size determination

For the first objective the sample was determined using single population proportion formula with considerations of 95% confidence level,5% degree of precision, 32% cessation from Debre Markos [28], with 10% nonresponse rate and the sample size is 369.

For the second objective the sample size was calculated by double population formula considering 95% confidence interval, 80% power, ratio of unexposed to exposed = 1, proportions obtained from a study done in Debre Markos town, Northern Ethiopia with P1 (proportion of cessation of breast feeding among women who were housewives, 25.4%) and P2 (proportion of cessation of breast feeding among women who were employed (government plus private), 38.5% [28]. The sample size obtained was 396. After adding 10% of potential non-response rate, the total sample size considered for this study was 436.

From the calculated sample sizes, the largest sample size to conduct the study is 436.

The sample mothers were selected using a mix of random sampling techniques. Initially, the list of total number of mothers with children aged 2 to 3 years at Arba Minch HDSS kebeles was obtained from the HDSS database. Then, a proportional allocation of mothers was made to each kebele based on the number of women in each kebele. The sample women eligible for interview were selected using a random selection from the list of women using the SPSS software select command.

### Variables of the study

Dependent variable of the present study is prevalence of early cessation of breast-feeding before two years. Independent variables were socio-demographic variables, feeding practice and related variables, obstetrics and related variables**.**

### Measurements

#### Early cessation of breastfeeding

Mother’s stoppages of breastfeeding before their children are two years of age [28].

#### Data collection tools and procedures

The quantitative data was collected using an interviewer-administered, pre-tested and structured questionnaire. The questions were adapted from women questionnaire on breast-feeding from the Ethiopian Demographic Health Survey and other related literatures from studies conducted on breastfeeding [1–3]. Thus, the questions will comprise socio-demographic, feeding practice, maternal and child health condition and obstetric determinants of early cessation of breast feeding and the data was collected from mothers with children aged two to three years. Wealth index of individuals household was assessed using an adapted questionnaire from the EDHS wealth index assessment tool. The questions were initially prepared in English then translated to Amharic and then back translated to English by language experts to ensure consistency. Thirteen HDSS data collectors who had prior experience in data collection were collected the data and four public health officers from the different district health facilities were assigned as supervisors. Two days long training was given to the data collectors and supervisors on the data collection process. The list of women to be interviewed from each kebele was provided to the data collectors in advance and health development army leaders guided the data collectors to the respective women to be interviewed on house-to-house basis. ODK application was used to collect data.

#### Data quality assurance

To assure the quality of data, thorough training was provided to the data collectors of. Before commencement of data collection, pretest was conducted on 5% of the sample size (44 mothers) in one of the kebeles in the district which was not part of the HDSS. Data was checked for completeness and consistency before data entry. The variables were coded, then edited during data entry. ODK was used to prepare template of questions and data was collected using the ODK mobile phone application. To minimize recall bias, intensive training was given for data collectors on about probing techniques using common local events.

#### Data analysis procedure

Data was initially entered into Epi-data software version 3.1 and then exported to SPSS version 25 statistical package for analysis. Descriptive analysis (frequencies, mean, proportion, standard deviation) was done and summarized by tables and graphs. Household wealth index (socio-economic status) of the individual households was analyzed by employing principal component analysis.

Bivariable logistics regression was used to select candidates’ variables. Variables with *P*-value <0.25 in the bivariable logistics regression model were entered into the multivariable logistics regression model in order to measure the association with the outcome variable after adjusting the effects of other variables using the Backward LR method. Variables with *P*-value<0.05 in the multivariable logistics regression analysis were considered as statistically significant for the cessation of breast-feeding before two years. Model fitness was checked by Hosmer-Lemeshow goodness fitness test. Multi-collinearity among explanatory variables was checked using Variance Inflation Factor (VIF >10). Odd ratio with its 95% confidence interval was reported to show the magnitude of association between the outcome and explanatory variables.

#### Ethical consideration

Ethical clearance was obtained from Institutional Review Board (IRB) of Arba Minch University, College of Medicine and Health Science. Respondents were informed about the purpose and procedure of the study and written consent was obtained from each participant. The written consent was obtained by the approval of the IRB for there could be participants who could not read and write. The privacy and confidentiality of the information was assured for participants in the study and the questionnaire was kept anonymous.

## Result

### Socio demographic characteristics

The response rate of the current study was 99.3% (433). Nearly two-third (66.3%) of the mothers was under the age group of 25–34 years. 76.9% of the mothers were protestant by religion and 79.9% were Gamo by ethnicity. Nearly all (98.8%) respondents were married and more than half (54.5%) were not have formal education. More-than one-third (37.9%) child fathers were not followed formal education. Nearly two third (65.80%) of the fathers were farmer by occupation. Regarding family size more than one-third (36.49%) had a family size of seven and above (Table 1).

**Table 1:** Socio demographic characteristics of participants in Arba Minch HDSS 2022

SN	Variables	Labels	Frequency	Percent
1	Mother age	15–24	46	10.6
		25–34	287	66.3
		35–44	87	20.1
		>45	13	3.0
2	Marital status	Married	428	98.8
		Single	4	0.9
		Divorced	1	0.2
3	Maternal Educational status	Illiterate	236	54.5
		Primary education	130	30.0
		Secondary education and above	67	15.5
4	Fathers educational status	Illiterate	164	37.9
		Primary education	191	44.1
		Secondary education and above	78	18.0
5	Maternal Occupation	Farmer	98	22.63
		Housewife	292	67.44
		Private	43	9.93
6	Father occupation	Farmer	285	65.8
		Government employee	33	7.6
		Unemployed	33	7.6
		Private worker	82	18.9

### Feeding practice and related factors

About one third (19.60%) of the respondent were lowest in wealth index. Nearly half (48.50%) of the mother got support from partner. More than two third (73.90%) of the mothers not listened radio. Half (50.35%) of the child’s were females. Among the reasons for early cessations of breast feeding, 29.30% of the mothers replied that they got pregnant. From the respondents 85.91% reported that they exclusively breast feed their child and 24.48% of the mothers bottle feed their child. Among the respondents 48.04% were provided practical breast feeding education (Table 2).

**Table 2:** Feeding practice and related factors of participants in Arba Minch HDSS 2022

SN	Variables	Labels	Frequency	Percent
1	Wealth index	Lowest	85	19.6
		Low	87	20.1
		Medium	96	22.2
		High	83	19.2
		Highest	82	18.9
2	Support from partner	Yes	210	48.50
		No	223	51.50
3	Child sex	Male	215	49.65
		Female	218	50.35
4	Exclusive breast feeding	Yes	372	85.91
		No	61	14.09
5	Bottle feeding	Yes	106	24.48
		No	327	75.52
6	Breastfeeding education	Yes	208	48.04
		No	225	51.96

### Feeding practice and related factors

**Fig. 1:**
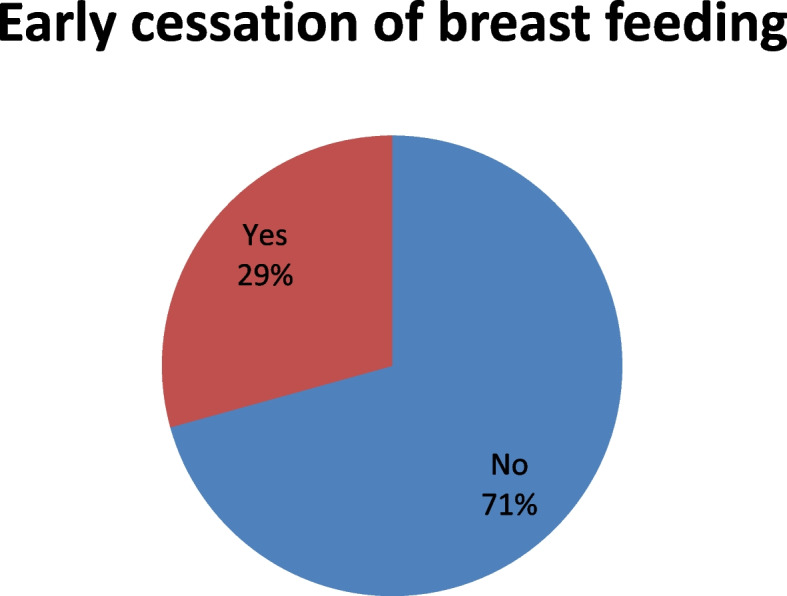
Early cessation of breast feeding of participants in Arba Minch HDSS 2022

Regarding breast feeding 29.3% had experienced early cessation of breast feeding (Fig. 1).

### Obstetric characteristics

For more than one-third (36.03%) of the mothers time to initiation of breast feeding were not the first hour. Regarding the age of the mothers at fist time of pregnancy, 75.29% were less-than 24 years of ages. Nearly one-third of the mothers were gave birth of more than five children. Half (49.74%) of the mothers were gave birth with interval of less-than 2 years. More-then one-third (37.88%) of the mothers were not followed antenatal care during their pregnancy of the index child. Nearly half (48.27%) of the mothers were delivered at home and majority (96.30%) of the respondents gave birth through spontaneous vaginal delivery (Table 3).

**Table 3:** Obstetric characteristics of participants in Arba Minch HDSS, 2022

SN	Variables	Labels	Frequency	Percent
1	Breast feeding initiation time	Within 1 hr	277	63.97
		>1 hr	156	36.03
2	Age at first time pregnant?	15–19	123	28.41
		20–24	203	46.88
		25–29	72	16.63
		> = 30	35	8.08
3	Number of births	1–2	146	33.7
		3–4	148	34.2
		> = 5	139	32.1
4	Birth interval(n = 386)	<=2	192	49.74
		>2	194	50.26
5	ANC	Yes	269	62.12
		No	164	37.88
6	Frequency of ANC (n = 269)	1st	15	5.58
		2nd – 3rd	126	46.84
		4th and above15	128	47.58
7	Frequency of PNC (n = 269)	Once	54	20.07
		Twice	60	22.30
		Three	155	57.62
8	Place of delivery	Home	209	48.27
		HC	163	37.64
		Hospital	61	14.09
9	Mode of delivery	Spontaneous vaginal delivery	417	96.30
		Caesarian section	16	3.70

### Factors associated with early cessation of breast feeding

Bivariable and multivariable logistic regression analysis was done to identify factors associated with early cessation of breast feeding. On the bivariate analysis, wealth index, father educational status, mother occupational status, family support, age at first pregnancy, birth interval, ANC visit frequency, place of child delivery, initiation of breast feeding showed *p*-value of <0.25 and became a candidate for multivariable analysis (Table 4).

**Table 4:** Multivariable logistic regression model showing factors associated with early cessation of breast feeding in Arba Minch HDSS, 2022

SN	Variables	Category	Early cessation of BF		COR (95%C. I)	AOR (95%C. I)	***P***-value
			Yes (%)	No (%)			
1	Wealth index	Lowest	21(24.7)	64(75.3)	1.74(0.81,3.76)	0.76(0.13,4.31)	0.76
		Low	26(29.9)	61(70.1)	2.26(1.07,4.79)	1.19(0.23,6.16)	0.84
		Medium	40(41.7)	56(58.3)	3.79(1.85,7.78)	1.54(0.29,8.08)	0.61
		High	27(32.5)	56(67.5)	2.56(1.21,5.42)	3.23(0.55,18.9)	0.19
		Highest	13(15.9)	69(84.1)	1	1	
2	Fathers educational status	No formal education	46(28.0)	118(72.0)	0.696(0.39,1.24)	0.22(0.07,0.74)	0.014
		Primary education	53(27.7)	138(72.3)	0.69(0.39,1.20)	0.30(0.12,0.76)	0.011
		Secondary and above	28(35.9)	50(64.1)	1	1	
3	Maternal occupational status	Farmer	55(56.1)	43(43.9)	4.22(1.87,9.51)	6.40(1.38,29.74)	0.018
		Housewife	62(21.2)	230(78.8)	0.89(0.42,1.90)	2.63(0.70,9.84)	0.15
		Private	10(23.3)	33(76.7)	1	1	
4	Family support	Yes	162(77.1)	48(22.9)	1	1	
		No	144(64.6)	79(35.4)	0.54(0.35,0.83)	1.69(0.75,3.83)	0.20
5	Age at first pregnancy	15–19	21(25.2)	92(74.8)	0.82(0.49,1.39)	0.76(0.32,1.82)	0.54
		20–24	44(21.7)	159(78.3)	3.51(1.89,6.50)	1.17(0.42,3.25)	0.77
		25–29	39(54.2)	33(45.8)	1.75(0.79,3.89)	1.05(0.32,3.25)	0.93
		> = 30	13(37.1)	22(62.9)	1	1	
6	Birth interval	<=2	82(42.7)	110(57.3)	3.51(2.19,5.6)	2.07(1.03,4.16)	0.041
		>2	34(17.5)	160(82.5)	1	1	
7	Frequency of ANC	1	5(33.3)	10(66.7)	1.57(0.50,4.93)	9.27(2.10,40.83)	0.003
		2–3	50(39.7)	76(60.3)	2.06(1.20,3.53)	2.73(1.27,5.88)	0.01
		> = 4	31(24.2)	97(75.8)	1	1	
8	Place of child delivery	Home	68(32.5)	141(67.5)	1.48(0.77,2.84)	1.46(0.45,4.72)	0.53
		Health center	44(27.0)	119(73.0)	1.34(0.58,2.23)	0.48(0.16,1.40)	0.17
		Hospital	15(24.6)	46(75.4)	1	1	
9	Initiation of breast feeding	Within 1 hr	69(24.9)	208(75.1)	1	1	
		>1	58(37.2)	98(62.8)	1.79(1.17,2.73)	1.77(0.74,4.24)	0.20

All variables had fulfilled chi-square assumption. On multivariable analysis, by taking other variables constant, child’s fathers with no formal education and primary education were 88 and 70% less likely to have early cessation of breast feeding as compared to secondary educated and above [AOR = 0.22; 95%CI (0.07,0.74)] and [AOR = 0.30; 95%CI (0.12,0.76)] respectively.

Maternal occupational status was also statistically significantly associated with early cessation of breast feeding. Farmer were 6.4 times more likely to early cessation breast feed than private occupation [AOR = 6.40; 95%CI: (1.38, 29.74)]. Those mothers with birth interval of less than 2 years were 2.07 times more likely to early cessation breast feed than those with birth interval of more than 2 years [AOR = 2.07; 95%CI: (1.03,4.16)].

Frequency of antenatal care was statistically significantly associated with early cessation of breast feeding. Mothers with one visit were 9.27 times more likely to early cessation of breast feed than those mothers with four visit and more [AOR = 9.27; 95%CI: ((2.10, 40.83)]. Similarly, Mothers with two to three visit were 2.73 times more likely to early cessation of breast feed than those mothers with four visit and more [AOR = 2.73; 95%CI: (1.27,5.88)] (Table 4).

Hosmer Lemeshow’s goodness-of-fit test produce chi-square of 2.962 with *p*-value of 0.94 and 8 degree of freedom hence the model was good for the data.

## Discussion

The study examined early cessation of breast feeding and factors affecting it in rural Southern Ethiopia.

In the study, more than one in four of women ceased breast feeding before 24 months of age of their children.

This finding is in line with the 2019 Ethiopian demographic and health survey national report [5]. However, it is lower than studies conducted in Abu Dhabi, United Arab Emirates [30] and Hula District, Southern Ethiopia [31]. Even though the two studies reported on early cessation of breast feeding, the populations considered were different. In the Abu Dhabi study, the participants were working women while they were rural women with children under one year for rural women in Hula district [30, 31]. Unlike the recommendation that children need to breastfeed up to the age of two years, proportion of women a high proportion of women in the study are lagging behind this in the current study. This might be explained by lack of adequate information about proper breast feeding practice by mothers in the present study, since about half (48.04%) of mothers in the current study did not attend breast feeding counseling. Exposure to such information gives an opportunity in achieving improved child breast feeding practice so that mothers could breast feed their child optimally [32].

The present study revealed that father’s educational status was statistically significantly associated with early cessation of breast feeding. Fathers who did not attend formal education and who attended primary education were less likely to early cease breast feeding as compared to those fathers who attended secondary education and above. The finding of the current study is consistent with a study conducted in Philippines. Fathers with higher education level were more likely to be involved in works requiring them to be away from their parenting child and might not have enough time to encourage the mother to breast feed the child optimally [33].

Occupational status was also statistically significantly associated with early cessation of breast feeding were more likely to early cease breast feeding than private workers. This is in line with a finding from Osun State, Nigeria. Moreover, previous study in Ethiopia documented a high sub-optimal breast feeding practice in the rural parts of the country which is predominantly populated with farmers [19, 31, 34]. This might be related to inadequate access to information on optimal breast feeding and health care owing to least access to health care facilities and health care professionals. Where adequate information might be available, the labor-intensive and time-demanding life in rural areas and farming work would deter farmers from adhering to recommendations of optimal breast feeding [31]. Tis might imply tailoring optimal breast feeding information to mothers from different job backgrounds in the study area.

Frequency of antenatal care was statistically significantly associated with early cessation of breast feeding. Mothers with three and less ante natal visit were more likely to early cease breast feeding than those mothers with four visits and more ANC attendance also showed association with breast feeding practice in previous studies from Ethiopia and other parts of the world [27, 31, 32]. In the current study, apart from ANC attendance, the frequency of attendance has also mattered for the association. During the later visits of ANC, there is a higher chance of getting advice on optimal breast feeding that might improve breast feeding practice [32]. It is clear that repeated contact to health professionals provides an opportunity to adhere to the optimal recommendation.

Short inter-birth interval has also association with early cessation of breast feeding. This finding is consistent with a systematic review done in Ethiopia [35]. This can be explained by the empirical shortage of time for feeding since women who give birth before two years gap in pregnancy would cease breast feeding because of the pregnancy itself. This implies the benefit of family planning for both children and mothers.

## Conclusion

More than one third of the mothers in the present study ceased breast feeding before twenty four months. Factors associated with early cessation of breast feeding were father’s educational status, maternal occupation, birth interval of less than two years and having less ante natal care visits than optimal.

Partner inclusive health education should be given for rural community including farmers about duration of optimal breast feeding. Antenatal care service provision should be strengthened so that mothers might have adequate number of visits. Counseling should be given regarding appropriate birth spacing and family planning. Moreover, breast feeding education should be enhanced among rural farmers in the study area, by extension to Ethiopia.

## Data Availability

The datasets used and analyzed during the current study are included in the manuscript.

## Abbreviations

CBF: Cessation of breast-feeding
EDHS: Ethiopian Demographic and Health Survey
HDSS: Health Demographic Surveillance Site
IQ: Intelligence Quotient
WHO: World Health Organization
UNICEF: United Nations Children’s Fund
